# Building locally anchored implementation science capacity: the case of the adolescent HIV implementation science alliance-supported local iS alliances

**DOI:** 10.3389/frhs.2024.1439957

**Published:** 2024-10-14

**Authors:** Susan Vorkoper, Kawango Agot, Dorothy E. Dow, Michael Mbizvo, Cyrus Mugo, Nadia A. Sam-Agudu, Fred C. Semitala, Brian C. Zanoni, Rachel Sturke

**Affiliations:** ^1^ Fogarty International Center, National Institutes of Health, Bethesda, MD, United States; ^2^ Impact Research and Development Organization, Kisumu, Kenya; ^3^ Duke Global Health Institute, Durham, NC, United States; ^4^ Department of Pediatrics, Infectious Diseases, Duke University Medical Center, Durham, NC, United States; ^5^ Population Council, Lusaka, Zambia; ^6^ Kenyatta National Hospital, Nairobi, Kenya; ^7^ International Research Center of Excellence, Institute of Human Virology Nigeria, Abuja, Nigeria; ^8^ Department of Paediatrics and Child Health, School of Medical Sciences, University of Cape Coast, Cape Coast, Ghana; ^9^ Global Pediatrics Program and Division of Infectious Diseases, Department of Pediatrics, University of Minnesota Medical School, Minneapolis, MN, United States; ^10^ Department of Internal Medicine & Makerere University Joint AIDS Program, College of Health Sciences Makerere University, Kampala, Uganda; ^11^ Division of Infectious Disease, Department of Pediatrics, Emory University School of Medicine, Atlanta, GA, United States; ^12^ Department of Pediatric Infectious Diseases, Children’s Healthcare of Atlanta, Atlanta, GA, United States; ^13^ Hubert Department of Global Health, Emory University Rollins School of Public Health, Atlanta, GA, United States

**Keywords:** implementation science, capacity building, adolescent HIV, Africa, collaboration, alliance

## Abstract

**Background:**

The Fogarty International Center-led Adolescent HIV Implementation Science Alliance (AHISA) supports region-/country-specific implementation science (IS) alliances that build collaborations between research, policy, and program partners that respond to local implementation challenges. AHISA supported the development of seven locally-led IS alliances: five country-specific (i.e., Kenya, South Africa, Tanzania, Uganda, and Zambia), one in Central and West Africa, and one with youth researchers. This article outlines the aims, activities, and outcomes of local alliances, demonstrating how they enhance sustainable IS activities to address local challenges.

**Methods:**

We conducted a desk review of each alliance's funding applications, reports, and data from the initial findings of a larger AHISA evaluation. The review analyzes common approaches, highlights their local relevance, and summarizes initial outcomes.

**Results:**

The local alliances have a common goal: to expand implementation of successful interventions to improve adolescent HIV. We identified four overarching themes across the local alliances’ activities: capacity building, priority setting, stakeholder engagement, and knowledge dissemination. Research capacity building activities include long-term mentorship between junior and senior researchers and short-term training for non-research partners. Setting priorities with members identifies local research needs and streamlines activities. Alliances incorporate substantial engagement between partners, particularly youth, who may serve as leaders and co-create activities. Dissemination shares activities and results broadly.

**Conclusion:**

Local IS alliances play a key role in building sustainable IS learning and collaboration platforms, enabling improved uptake of evidence into policy and programs, increased IS research capacity, and shared approaches to addressing implementation challenges.

## Introduction

Reducing the rates of adolescent HIV hinges on the successful implementation of effective interventions to decrease transmission and improve treatment outcomes. It calls for tailored, evidence-based approaches to address the unique needs of adolescents and responds to the specific context in which they live. Implementation science (IS) holds promises for addressing these challenges by improving the understanding of barriers to health programming, effective implementation strategies, and facilitators of adoption and uptake of interventions that work (1).

The Adolescent HIV Implementation Science Alliance (AHISA), a learning collaborative made up of a network of researchers, policymakers, program implementers, and youth that promotes the use of IS to enhance uptake of evidence and overcome implementation challenges related to the prevention and treatment of HIV among adolescents in Africa (2). AHISA is comprised of 26 teams of NIH-supported implementation researchers and their in-country partners working across 11 African countries. To bolster sustainability and local relevance, AHISA supports locally led projects aimed at decentralizing knowledge creation, building IS capacity, promoting equitable partnerships, and contributing to the sustainability of IS activities (3). The overall objective of supporting these projects is to develop a learning ecosystem for IS activities that link available IS research evidence to local practice and policy, thereby making the science and its outcomes more accessible, locally relevant, and potentially sustainable.

Since 2019, AHISA has supported the development of seven country- and region-specific IS alliances anchored in building collaboration between research, policy, and programs that are able to respond to local implementation and IS research challenges. Five are country specific- Kenya (KAHISA), South Africa [AHI(SA)^2^], Tanzania (TAHISA), Uganda (UAHISA), and Zambia (ZAHISA)- one focuses on the Central and West Africa region (CAWISA), and one is centered on youth researchers in East and Southern African (African Youth IS Alliance, AYISA). These alliances leverage the AHISA model and represent an investment aimed at building sustainability and responsiveness to local implementation and implementation science challenges and needs. These local alliances catalyze various activities that are aligned with local priorities, programmatic needs, and policy questions thereby creating unique opportunities to scale-up and sustain the implementation research evidence generated by network members.

This approach is modeled on the successful Nigeria Implementation Science Alliance (NISA), a collaborative IS consortium of 20 local organizations, universities, and government agencies in Nigeria (4). NISA was established in 2015 and was an outcome of the Fogarty-led NIH-PEPFAR Prevention of Mother to Child Transmission (PMTCT) Implementation Science Alliance (5). NISA meets annually, with attendance at each meeting by the federal and state health personnel, including the Nigerian Minister of Health. NISA represents an important and self-sustaining extension of the work of the PMTCT Alliance and provides a platform for Nigerian researchers, program implementers, and policymakers to identify and/or generate evidence and implementation strategies that address critical local implementation challenges (6).

Adapting this model, each of the seven AHISA local alliances identifies country and regionally specific implementation issues and seeks innovative solutions through dynamic learning platforms that leverage IS methods. They convene partners to develop priorities, discuss successes and challenges in implementation, set agendas for alliance activities, and share research results. By encouraging a local focus, these alliances are more nimble and agile than the larger AHISA network in addressing local policies and priority health areas in their focus country/countries.

This article describes the aims, activities, outputs, and outcomes of the local alliances to illustrate how they enhance the potential for sustainable IS activities that address local implementation challenges. It explores their unique approaches and reflects on the role that country- and regional alliances can play in the future of IS.

## Methods

Data was gathered from desk reviews of applications, final reports, and documents from April 2019 to January 2024, alongside a preliminary AHISA evaluation outlining local alliance deliverables, outcomes, and impacts. Two authors (SV, RS) coded the documents by hand in excel using a codebook developed based on discussions held at the AHISA annual meetings on the commonalities and themes across the alliances and in response to the key research questions (Appendix 1) to extract the relevant information related to their goals and activities, the role of IS, and their engagement practices. Discrepancies in the data were discussed until there was a consensus. One author (SV) did an initial analysis of the final data to create broad themes; these were shared and discussed by all authors to further refine and decide on key examples. The review provides an analysis of the commonalities between the alliance approaches, illustrates their relevance to the local context, and contributions to sustainable IS activities.

## Findings

All seven AHISA local alliances have a common overarching goal: to expand the implementation of successful interventions focused on improving HIV prevention and care. A key principle of each of the alliances is to foster dialogue and learning between researchers, HIV program implementers, youth, and policymakers. Additional critical priorities of each alliance include building IS capacity, identifying evidence-based interventions to support response strategies, and enhancing translation of evidence into policy and practice. All the alliances are led by a leadership team comprised of AHISA members, IS experts, and other invested parties that meet regularly to ensure alliance activities continue to meet local needs and are achieving their intended outcomes.

**Figure 1:**
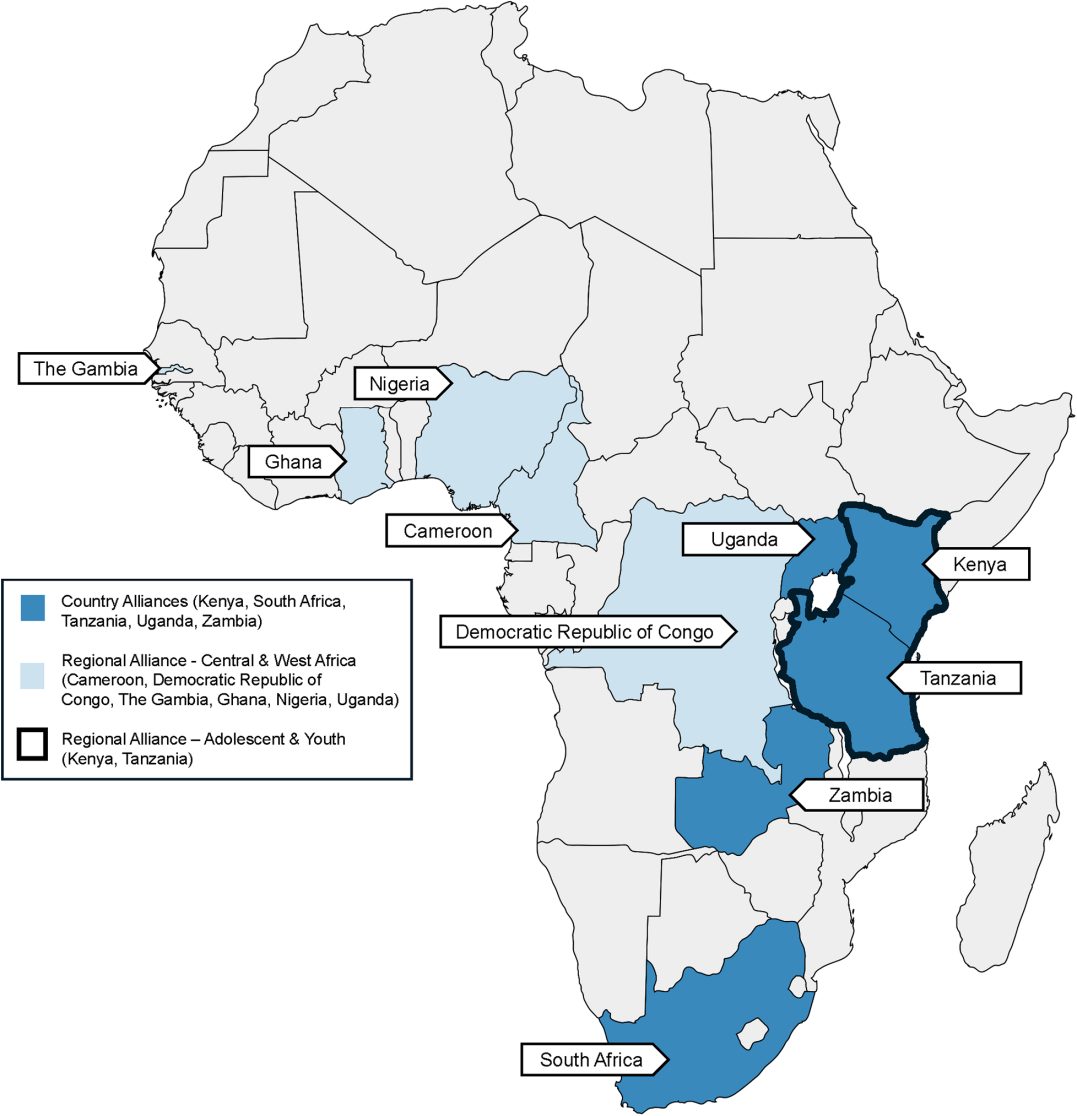
Map of local AHISA alliances.

Though the overall goal is the same, their models and activities differ (Table 1. AHISA Local Alliances’ Goals and Activities; Figure 1. Map of Local Alliances). For instance, some are focused on responding to specific Ministry of Health policy questions (ZAHISA), while others are focused on creating a platform for learning and communication among IS researchers [AHI(SA)^2^] or prioritize supporting research independence and have created long-term mentorship for local researchers (CAWISA). All of the alliances are nimble and responsive to evolving local needs. This is illustrated in their evolving foci and activities over time as they, for example, push into new provinces or expand their alliance to include new partners. The following section describes the findings from a review of their activities and outcomes across four common categories: (1) capacity building; (2) priority setting; (3) dissemination; and (4) engagement practices.

**Table 1:** Summary of AHISA local alliances’ goals and activities.

Local alliance	Countries	Years	Description	Alliance aims	Major activities
Country alliances					
AHI(SA)^2^: Adolescent HIV Imp Science Alliance—South Africa	South Africa	2019, 2020, 2021	The South African Adolescent HIV Implementation Science Alliance [AHI(SA)2] is a group of key stakeholders involved in adolescent HIV care, treatment, prevention and research in South Africa. The goals are to exchange ideas, identify challenges and create creative solutions to implementing interventions along the continuum of prevention and treatment of HIV among adolescents. They aim to work with youth to create approaches to implementation science and work together toward collaborative solutions.	(1) Create a network of researchers, policymakers, implementers, clinicians, community leaders, and adolescents working on the adolescent HIV continuum of care in South Africa to collaborate, share resources and ideas, share successes and failures of intervention development and implementation; (2) expand implementation of successful interventions focused on improving each step along the adolescent HIV continuum of prevention and care; (3) create collaborative research teams to design interventions addressing adolescent HIV prevention, and the adolescent HIV continuum of care in SA	Used a modified Delphi technique to create expert consensus to leverage policymakers on the use of evidence-based interventions to improve the adolescent HIV continuum of care in South Africa, and to conduct a systematic review of interventions designed for adolescents living with HIV along each stage of the continuum of prevention and care in South Africa. Based on these findings, held virtual and in-person meetings on a range of topics, including continuum of care, prevention, and community engagement. Created collaborative working groups on HIV prevention, adolescent-friendly services, HIV testing and linkage to care, retention and adherence, and transition to adult care. Established a youth leadership training program and developed a youth research advisory board with youth who went through the training.
KAHISA: Kenya Adolescent HIV Imp Science Alliance	Kenya	2019, 2020, 2021, 2023	The Kenya Adolescent HIV Implementation Science Alliance (KAHISA) aims to build capacity for implementation science among stakeholders working in the field of adolescent and young adult HIV in Kenya. KAHISA is training 20 program implementers in five high HIV burden counties in Kenya on implementation science. KAHISA is leveraging resources available in Kenya to train and support small grant implementation science work in these counties including the Kenyatta National Hospital online training platform, the University of Nairobi trainees, and the ACQUIRE Quality Improvement training platform, which aims to promote evidence-guided quality improvement for frontline health workers and institutions.	Bring together national policymakers, implementing partners, researchers, other stakeholders to (1) build implementation science support and coordination; (2) facilitate implementation science research capacity among stakeholders involved in adolescent HIV work; (3) support formulation and implementation of strategies aimed at halting the HIV pandemic among AYA, (4) disseminate findings of these efforts by presenting findings to community, scientific and policy audiences and publishing in reputable journals, and (4) improve outcomes among AYA living with HIV.	Held in-person and virtual meetings and symposiums with stakeholders in response to Kenya's Fast-Track Plan to End HIV and AIDS among Adolescents and Young People policy and other areas of interest to the alliance membership; held five-day didactic implementation science training with stakeholders from five high HIV burden counties in Kenya and two trainings for other KAHISA members; conducted and published a systematic review
TAHISA: Tanzanian Adolescent HIV Prevention and Treatment Imp Science Alliance	Tanzania	2020	The Tanzanian Adolescent HIV Prevention and Treatment Implementation Science Alliance (T-AHISA) goals are to formally establish a sustainable network of key Tanzanian stakeholders (e.g., researchers, implementors, policy makers, and youth advocates) engaged in adolescent HIV-focused research and programs in Tanzania; to develop an implementation science research agenda with the Tanzanian government to help tackle the most pressing challenges leveraging the robust in-country network; and to build capacity for implementation science research by mentoring early stage investigators with paired mentors from the alliance.	To formally establish a sustainable network of key Tanzanian stakeholders (researchers, implementors, policy makers, and youth advocates) engaged in adolescent HIV-focused research and programs in Tanzania; to develop an implementation science research agenda with the Tanzanian government to help tackle the most pressing challenges leveraging this robust in-country network; and to build capacity for implementation science by mentoring early stage investigators with paired mentors from the alliance.	Held inaugural meeting on the importance of adolescent mental health in HIV prevention and treatment; developed mentee-mentor adolescent HIV implementation science research dyads and created broader East-Africa research “pods”; established formal youth community advisory board in four study regions and incorporated chairpersons of the youth CABs within the current T-AHISA network; complete a concept mapping exercise to identify the critical factors that impact the implementation of HIV-related prevention and treatment programs for Tanzanian youth from the youth perspective
UAHISA: Uganda Adolescent HIV Prevention and Treatment Imp Science Alliance	Uganda	2019, 2020, 2021, 2022	The Uganda Adolescent HIV Prevention and Treatment Implementation Science Alliance (U-AHISA) promotes the use of evidence-based interventions to deliver HIV services to adolescents and young people in Uganda by bringing together officials from government ministries, academia, youth, and HIV implementing programs. After being trained on implementation science core principles and how it related to the gaps along the continuum of HIV services, these stakeholders have worked in small, thematic groups, including mental health, Antiretroviral Therapy (ART) & Viral Suppression, and HIV testing services, to identify gaps, develop interventions to address those gaps, mentor HIV-positive adolescents, share findings, and develop implementation science research agendas that are used to apply for research funding.	To improve coordination, build implementation science capacity, and identify evidence-based interventions to support response strategies in both the community and clinical settings by bringing together multisectoral partners involved in implementation of HIV services and those receiving these services, with youth participation being *a priori*ty. To leverage existing Ugandan Ministry of Health mechanisms to decentralize capacity building for implementation science to regional public health facilities.	Formed multi-disciplinary and multisector small groups to drive adolescent centered evidence-based interventions to review, synthesize and document evidence-based interventions, develop strategies to adopt and adapt evidence-based interventions that address identified implementation gaps through capacity building and mentorships in Implementation Science, and identify a list of priority research questions along the HIV continuum of care for the ALHIV. Engaged stakeholders in the design and implementation stages to promote uptake of evidence-based intervention. U-AHISA implemented project within two early adopter regional hospitals activities include: project entry meetings to introduce U-AHISA, inaugural meetings, provide trainings, conduct a needs assessment at both sites, form regional thematic teams, and hold six regional alliance meetings. Expanding effort to an additional regional hospital. Promote the use of evidence-based interventions in the delivery of HIV services for ALHIV through regular interactions of U-AHISA with HIV program implementers.
ZAHISA: Zambia Adolescent HIV Prevention and Treatment Imp Science Alliance	Zambia	2019, 2020, 2021, 2022	The Zambia Adolescent HIV Prevention and Treatment Implementation Science Alliance (ZAHISA) is a local platform for sharing related research activities and findings. The alliance has been invaluable in their efforts to work with adolescents and strengthening implementation science research capacity for HIV prevention and care, identify barriers to accessing services, and participate in research. ZAHISA has further ensured youth engagement through their participation in the ZAHISA Advisory Committee, which is chaired by a Director in the Ministry of Health. ZAHISA is collating and documenting lessons, challenges and success stories, and establishing preferences for addressing stigma and discrimination associated with access and utilization of HIV testing and treatment services and research among adolescents and young adults.	Develop a collaborative forum to bring together multi-sectoral partners involved in implementation science research for improving HIV services and access, with youth participation being *a priori*ty, to create a dialogue on implementation science research priorities for Zambia	Convened a symposium to bring together multidisciplinary stakeholders to share ongoing research and provide a forum for building implementation capacity through peer-to-peer support; developed adaptations to AHISA-related research in light of COVID-19 and shared highlights from implementation science research findings and information on promising interventions and programmatic approaches among members and at the December 2021 national adolescent health symposium. Included youth between 18 and 24 years in a study as key informants and program managers. Held a series of six in-person workshops and member surveys to assess individual experiences and internalized HIV stigma concerns, identify strategies in place for addressing stigma among research studies/programs involving YPLHIV, and explore AYA perspectives and preferences on reducing HIV related stigma to enhance access to and utilization of HIV testing services and participate in research
Regional Alliances					
AYISA: African Youth Imp Science Alliance	East and Southern Africa	2019, 2021	The African Youth Implementation Science Alliance (AYISA) is anchored on the Reducing HIV in Adolescents and Youth Conference and its 30-under-30 initiative where people under 30 years old submitted concept proposals on reducing HIV among youth ages 15–24 years in Eastern and Southern Africa. The concepts were competitively selected by youth and experts. The 30 winners were mentored by senior researchers to develop their concepts into full protocols and awarded seed funding to pilot their projects. The 30 winners are the founding members of AYISA. They are being trained on the basics of implementation science and collecting implementation data as part of their pilot work.	To develop a cadre of young researchers and build their capacity in implementation science to address HIV; to conduct an IS training and develop subsequent networking opportunities for young researchers who receive 30-Under-30 awards, funded by the Bill and Melinda Gates Foundation, and provide ongoing mentorship on IS to awardees to ensure they maintain fidelity to the IS designs and frameworks they choose.	Identified and trained 30 young research from the Reducing HIV in Adolescents and Youth Conference (RHAY) on implementation science methods and frameworks; created an RHAY implementation science expert committee to guide overall efforts and serve as mentors; mentees presented preliminary results of their 30-under-30 data; held two-week AYISA workshop to support data analysis and drafting implementation science manuscripts for publication.
CAWISA: Central and West Africa Imp Science Alliance	Ghana, Nigeria, Cameroon, Democratic Republic of the Congo	2019, 2020, 2023	The Central and West Africa Implementation Science Alliance (CAWISA) is a regionally-focused consortium of senior researchers and postdoctoral mentees in multidisciplinary biomedical and behavioral research. CAWISA aims to build durable local implementation science capacity to address major public health issues in West and Central Africa, including adolescent HIV. The five-point sustainability plan includes the following: (1) Leadership and Vision; (2) Scholars and Mentors: engaging individuals committed to scientific advancements, diligent in scholarship and integrity, and willing to continuously pay it forward; (3) Strategic Partnerships; (4) Research Productivity and Impact and (5) Sustained Funding.	(1) Establish a Central and West African Region implementation science alliance and affiliated Centers of Excellence to generate regional evidence for HIV prevention and control among adolescents and young adults; (2) train and mentor CAWISA scholars to conduct implementation science research, successfully apply for funding, and write and publish data-driven manuscripts; (3) implementing short, on-site IS training sessions for young investigators and faculty	Select XX scholars and pair them with two mentors: one from core CAWISA team and one from a local institution for 2–5 years (approximately through the life of a grant led by scholar); develop and standardize a locally tailored implementation science career development toolkit to facilitate implementation science capacity and skill building as well as grantsmanship self-efficacy for early investigator mentees; develop and host implementation science training for each of the centers of excellence; conducted systematic review on HIV prevention and treatment for adolescents and young adults and systematically collate and publish cross-CAWISA country data on lessons learned and innovations applied in context of pandemic-adjusted HIV programming for adolescents and young adults

### Capacity building

Aligned with the overall AHISA goal of building IS capacity, five of the local alliances engage in robust capacity building activities. A key common component of these efforts is support for mentorship, pairing junior researchers with more established implementation scientists. For instance, AYISA partnered with members of the Fogarty-funded *Improving the HIV Care Cascade in Kenya through Implementation Science Training* (7) at Kenyatta National Hospital to provide training and mentorship to young research mentees from East and Southern Africa who were selected to join the Gates-supported 30-under-30 research program as part of the Reducing HIV in Adolescents and Youth (RHAY). AYISA and Kenyatta National Hospital offered stand-alone IS training to young people and also provided IS training as part of the 2022 RHAY conference. Each of the 30 winners meets regularly with a senior researcher who mentor them to develop full protocols, obtain approvals from ethics and other regulatory bodies in their respective countries, pilot their projects, and write up the results for peer-reviewed publications. As a result, the scholars added IS objectives to their study designs allowing them to collect implementation data that would inform policy considerations for projects that show impact.

Local alliances also commonly provide short-term training opportunities in IS. Many of the trainings included alliance members new to IS, including non-research partners from the Ministry of Health and program implementing organizations (KAHISA, UAHISA) and junior researchers (CAWISA), or trained youth as a lead up to developing a youth research advisory group [AHI(SA)^2^]. To ensure local relevance, several of the alliances develop and tailor training materials to the specific context by adapting existing trainings (KAHISA), developing new curriculum (UAHISA), and/or working with local partners on the content and delivery (CAWISA). The capacity building efforts sometimes lead to a specific outcome or an activity that allows the new members to apply their learning and advance the goals of the local alliance. For example, as part of a series of monthly trainings, UAHISA sub-groups developed a list of priority research questions through a process of identifying implementation gaps and potential evidence-based interventions related to their respective thematic areas along the HIV continuum of care for adolescents and young persons.

In many cases, training is paired with mentorship opportunities, enhancing the overall learning experience for participants. For instance, CAWISA pairs postdoctoral mentees in multidisciplinary biomedical and behavioral research with two senior research mentors. The CAWISA mentees are trained and mentored to conduct IS research, successfully apply for funding, and write and publish data-driven manuscripts. The CAWISA leadership team co-developed an IS curriculum content and learning objectives tailored to Central and West African country contexts and assembled an online, open-access ScholarIS toolkit that is pending release. As part of the training, mentees brainstorm small IS projects to work on with their mentors and are put into collaborative writing groups consisting of mentors and mentees to further learn from each other.

IS training fosters a common language that enables researchers and non-researchers to collaborate more effectively on challenges, understand the potential of IS to address those challenges, and leverage the evidence generated. For example, KAHISA led an online IS training for the Ministry of Health and HIV implementing partners from five counties with the highest HIV burden in Kenya, including Kisumu, Homabay, Siaya, Nairobi and Migori. With input from the University of Washington, the team adapted the training curriculum from one developed by the Department of Research and Program at the Kenyatta National Hospital. In total, 20 participants, identified by their respective county chief officers of health, took part in the training. After, they were paired with a local mentor to develop IS proposals that assessed the feasibility of proposed interventions and implementation strategies. Projects ranged from adherence counseling for adolescents with viral failure to quality improvement of Pre exposure prophylaxis (PrEP) to a transition readiness assessment for adolescents moving to adult care. Along the way, trainees participated in KAHISA-led symposiums to share results. They also continued their learning through trainings on dissemination strategies including how to develop policy brief and engaging presentation at scientific and non-scientific forums.

### Priority setting

A major part of the local alliance approaches has been identifying priority areas to utilize IS to answer pressing questions related to the implementation of proven interventions for adolescent HIV. This leverages the collective wisdom of the group and their multiple perspectives. Most of the priority setting is done in the lead up to deciding on alliance activities, and many have continued to reflect on key areas as the HIV context evolves. Some have also expanded these efforts to new locations (as UAHISA did when they grew from the capital city to new regional referral hospitals and program implementers) or additional health areas considering new developments. For example, ZAHISA held symposiums as part of national health research conferences and workshops on understanding trends in service utilization and access during COVID, as these needs and opportunities arose. It has also facilitated learning between partners and youth engaged in IS activities through national mapping of IS research and giving youth, including those living with HIV, a voice. Other alliances focused on specific topics within the larger adolescent HIV IS field, developing themes and leveraging the unique perspective and expertise of alliance members to address the themes. For example, UAHISA created thematic groups based on members’ interests and expertise that looked at challenges and opportunities to use IS across the HIV continuum of care specific to adolescents. These groups have met regularly for training and to identify evidence-based interventions and successful implementation strategies. In addition, they hold partnership meetings with USAID, CDC, and community health departments to consider how to integrate their findings into routine care.

Priority setting approaches have varied from the utilization of systematic DELPHI [AHI(SA)^2^] (8) and study mapping (KAHISA) to surveys and key informant interviews (ZAHISA) and to workshops that gather real time input from their multi-sector members (TAHISA). These activities involve substantial engagement with their members, providing opportunities to analyze gaps, identify existing evidence-based interventions, and develop implementation research questions. Given the nature of the alliances’ engagement with non-research partners, these identified priorities are often more locally grounded in policy and programmatic context.

### Dissemination

There is recognition across the local alliances that disseminating alliance learning is key to ensuring that research evidence is used to inform policy and programs. Some alliances provided specific training on dissemination to non-research audiences including how to write a policy brief with practice presenting to Ministry of Health officials (KAHISA). Others meet with stakeholder groups beyond the alliance membership to ensure their engagement (UAHISA). Many share results and on-going activities among their members, both in-person and virtually, allowing them to learn from each other and ensure they are working towards the goals of the alliance. There is also a substantial emphasis on training early-stage investigators to write publishable manuscripts including CAWISA that developed writing groups to support each other's efforts and AYISA that is holding virtual meetings with the RHAY 30-U-30 awardees on manuscript writing.

Dissemination activities are often driven by priority setting efforts. For instance, TAHISA held an initial research priority setting workshop with researchers, implementers, policymakers, and youth advocates to identify local needs around adolescent mental health and HIV and the role research can play in addressing those issues. Along with developing alliance objectives, they highlighted the need to incorporate youth in the decision making around any activities that concern them. As a result, a youth advocate presented at the Tanzania Commission for AIDS sub-group on adolescent and gender in reproductive health and continues to represent the alliance at relevant meetings and conferences. Members of AYISA have also presented their pilot work at local and international conferences, with one published in a peer-reviewed journal (9) and others at various stages of development.

### Engagement practices

The alliance model is premised on the concept of catalyzing and supporting communication, collaboration, and learning between researchers and users of research evidence including youth, ministry officials, and program implementers, among others (Table 2. Engagement Efforts Across the Local AHISA Alliances). Some local alliances have integrated these groups into the leadership of the alliance while others started with researchers and have expanded engagement to include youth and policymakers not originally part of the group. Beyond leadership teams, diverse community groups have joined and actively participated in collaborative working groups.

**Table 2:** Engagement efforts across the local AHISA alliances.

Local alliance	Research collaborations	Collaborations with Ministry of Health Officials	Collaborations with clinicians	Collaborations with HIV program implementers	Collaborations with youth
AYISA	X				X
CAWISA	X				
KAHISA	X	X	X		X
AHI(SA)^2^	X		X		X
TAHISA	X			X	X
UAHISA	X	X	X		X
ZAHISA	X	X		X	X

Government ministries, particularly ministries of health, have played an active and sometimes leadership role in six of the alliances. For instance, the Ministry of Health Assistant Director in charge of Adolescent Health chairs the ZAHISA Technical Advisory Committee, which also includes a youth advisory panel, that identifies gaps and needs and reviews and provides guidance on the alliance's effort. The youth voices through this engagement have also helped to identify barriers to accessing services by youth, which helps to further shape IS research questions. The County Government of Kisumu's Ministry of Health helped plan for and offered outreach services during the initial RHAY Conference which AYISA was part of. Ministries, both federal and local, are also a key group that alliances have targeted for dissemination. This has included invitations to workshops and symposiums as well as special outreach specifically to these groups. For instance, TAHISA has sent members to participate in ongoing Ministry of Health groups and KAHISA had training to develop materials for stakeholder groups including Ministry of Health and local community. UAHISA has developed three regional groups of health care providers and a USAID-funded program implementing partners and paired them with IS mentors to conduct activities related to thematic areas identified by the regional alliance members.

Along with the policymakers, many of the alliances emphasize the importance of including youth throughout their efforts. Across many alliances, youth are active and fully engaged members. AHI(SA)^2^ first trained ten youth in research methodology, ethics, and IS. These youth now act as an advisory board to researchers, implementation scientists, and community programs as they develop new proposals or implementation of new programs. AHI(SA)^2^ members and their colleagues are encouraged to submit grant proposals, research protocols, or intervention manuals to the youth advisory board for input and feedback. AYISA also had youth serve as reviewers in their proposal selection process and engaged an all-youth secretariat to guide the awardees through all 30-U-30 processes, while TAHISA has engaged youth as equal partners in the alliance and invited youth collaborators to be part of the Tanzania commission for AIDS. TAHISA worked with their youth community advisory boards across four different Tanzanian regions to prioritize health challenges faced by young people and identify their recommended strategies and solutions. These findings were presented by youth to the Tanzania Ministry of Health and published in Frontiers of Public Health in 2024 (10).

The local alliances have also used the larger AHISA annual meetings and activities to learn from each other and further build relationships and collaboration. Each year at the annual AHISA forum, the local alliances share their aims and activities and have an opportunity to discuss challenges. They report that this forum has been key to exploring new possibilities and refining their work as well as developing new research projects. For example, a collaboration between UAHISA and KAHISA investigators resulted in the development of a new research proposal.

## Outcomes

**Figure 2:**
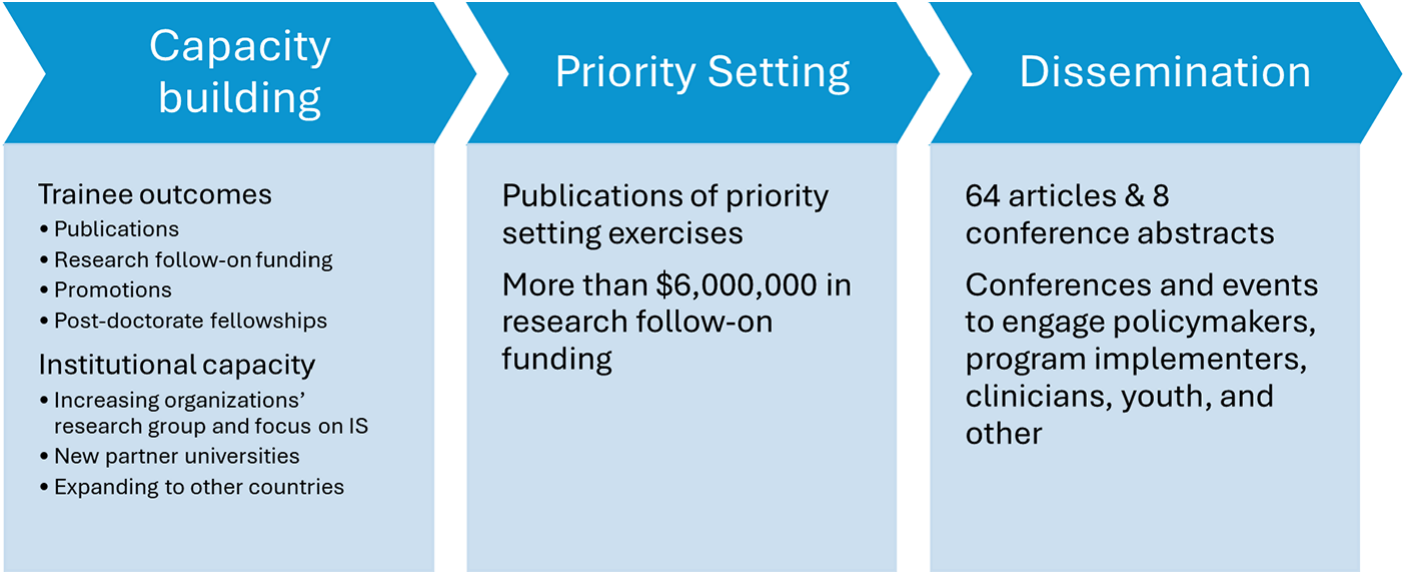
Key accomplishments off the local HIV implementation science alliances.

There are already several exciting outcomes from the local alliances demonstrating their value to building capacity for IS, using IS to address local challenges, and anchoring sustainable activities locally (Figure 2. Key Accomplishments of the Local HIV Implementation Science Alliances).

### Capacity building outcomes

In total, the local alliances report training 134 people. Of those, over a third were youth (35.8%) and about a third were health policy administrators (29.9%). Over 70% of these were trained through the local alliances in short-term training in contrast to the long-term mentorship programs, like CAWISA, that included only eight trainees. One member of KAHISA conducted a quality improvement project under KAHISA mentorship that was recently accepted for a poster presentation and registration scholarship at the International AIDS Conference in 2024. All 30 of the AYISA trainees went on to conduct their pilot studies. While many are still analyzing data, one recently had their manuscript published in PLOS Global Public Health (9) and over 10 abstracts have been presented at international conferences. Another worked with their mentor to receive a research planning grant from the NIH National Cancer Institute on using electronic reminders for HPV vaccinations (11). Trainees have advanced their careers with one receiving a scholarship for a master's program after highlighting the research capacity they built through RHAY in their application, and another recently accepting a new position as the County adolescent youth program's HIV coordinator. CAWISA reports that their trainees have received promotions to become senior members of their organizations, including one who became senior project officer in charge of accountability at an NGO and another who was promoted to Associate Professor and Head of Department for the School of Public Health. One has continued their training, as he was recently accepted for a post-doctorate fellowship at Yale University.

For CAWISA, the mentoring junior investigators (doctoral students and postdoctoral fellows) approach is being adopted in their partner universities. In fact, CAWISA was recently invited to expand their efforts to the Gambia. The alliance is now creating short-term, tailored trainings in IS to further introduce the approach to others across West and Central Africa. The last half day of the training is devoted to brainstorming small IS projects with a group of motivated training participants that will seed further efforts. Through their capacity building efforts, AHI(SA)^2^ reports that the University of KwaZulu Natal has expanded the size of their research group from three to 13, including nurses and clinical trial supervisory personnel, and are beginning to conduct new collaborative pediatric clinical research studies using IS research methods.

### Priority setting outcomes

All seven local alliances identified follow-on research stemming from their efforts, specifically successfully receiving over $6,250,000 in additional funding to support predominantly research proposals. Three of these awards were from the US National Institutes of Health. One is an investigator-initiated research grant, entitled “Interactive transition support for adolescents living with HIV comparing virtual and in-person delivery through a stepped-wedge cluster randomized clinical trial in South Africa” (8), that leverages the work of AHI(SA)^2^ to test mHealth interventions using a hybrid effectiveness- implementation design. In addition, as a result of AHI(SA)^2^ priority setting, the PIs successfully competed for a five-year, cooperative agreement “Evaluation of Long Acting Injectable and Teen Clubs in adolescents (ATTUNE)” (12). In addition, CAWISA PIs received a training grant to expand the pool of independent investigators in IS in Nigeria that builds off the CAWISA model (13). CAWISA also received funding to develop their IS career development toolkit to support researchers and practitioners in West and Central Africa in applying for implementation science funding. The other UAHISA members successfully applied for a grant from the International Association of Providers of AIDS Care to assess the effectiveness and outcomes of dispensing messages on adherence and viral suppression among children with an unsuppressed viral load in Uganda, a research gap identified by the alliance members. In addition, alliances received funds to pilot research studies conducted by trainees.

### Dissemination efforts outcomes

The local alliances report publishing over 60 peer-reviewed journal manuscripts and were part of eight international and local conference presentations/posters as an outcome of their work. This includes a systematic review and DELPHI analysis on interventions addressing the adolescent HIV continuum of care in South Africa (14), a reflection on provider-led adaptations to mobile phone delivery of the Adolescent Transition Package in Kenya (15), and a look at health worker perceptions of stigma towards Zambian adolescent girls and young women (16). Many of these were the result of new collaborations developed by the alliance, including publications with mentees and alliance members as first authors with the local alliance PIs and other steering committee members as mentors. In addition, the alliances also published newsletters and presented at international conferences both with poster and podium presentations. These are outputs above and beyond the conferences or events hosted by the local alliances themselves. While many of these alliance efforts are too new to have demonstrated long-term health impacts, UAHISA reports that their thematic group working on tuberculosis prevention in HIV/AIDS patients has been included in Uganda's guidelines.

## Discussion

In fostering sustainable, long-term collaborations among researchers, clinicians, policymakers, implementers, and youth, the local alliances have played a key role in building sustainable and nimble learning platforms. They have contributed to critical increases in IS capacity. Specifically, training allowed the alliances to build IS capacity and orient new members to what for many was a new field. These efforts establish a shared framework and understanding of IS among diverse partners. For the country alliances specifically, as their efforts progressed, they often provided additional training opportunities developed and tailored to the unique needs of their members. In contrast, the two regional alliances were focused almost exclusively on capacity building- investing much of their work in developing and maintaining long-term mentorship programs.

In addition, the learning spaces the alliances create provide unconventional but critical engagement of non-researchers that enhance dissemination, overall capacity, and enable a stronger response to critical challenges. Specifically, non-researchers are invited to not only learn about and provide input on alliance activities, but also lead efforts. While the approaches to engagement are unique to each alliance and its goals, the overarching engagement constructs, including partnership exchange, capacity building, and collaboration, are common across them (17).

This study has several limitations. First, this is a self-evaluation of the activities, themes, and outcomes of the local alliances, which presents a limited perspective and is inherently prone to bias. Second, this is not an exhaustive review of the full range of unique activities of each alliance but is rather intended to highlight key points. Finally, this work is limited to AHISA-supported local alliances focusing on adolescent HIV and does not address other collaboration models.

Additional broader limitations to this manuscript and analysis include an inability to assess how sustainable these alliances will be given how young they are. Future analyses should include metrics to assess sustainability. Moreover, the analysis was not framed around key gaps or challenges in implementation and how the local alliance helped to address these challenges and whether their efforts were successful, which future analyses could also include.

The AHISA local alliances lay the foundation for sustaining IS activities in high-burden HIV countries by anchoring research agendas in locally identified challenges and goals. By engaging policymakers, researchers, HIV practitioners, and youth from the community, the alliances ensure that research priorities and solutions are responsive to local needs. The AHISA local alliances epitomize a collaborative, community-driven learning ecosystem that offers invaluable insights and best practices for informing IS research-to-action endeavors worldwide.

## Data Availability

The datasets presented in this article are not readily available because it is not possible to anonymize the qualitative data from such a small data set that is specific to each country or regional alliance. Requests to access the datasets should be directed to Susan Vorkoper, susan.vorkoper@nih.gov.

## In memoriam

Since the submission of this publication, Dr. Kawango Agot has passed away. On behalf of the Adolescent HIV Implementation Science Alliance, we wanted to acknowledge her as a tireless mentor and advocate for youth. The impact of her work will be felt for years to come.

## APPENDIX 1

### Codebook for portfolio analysis of local implementation science alliances

Goal: to understand the unique and common approaches across the AHISA local alliances; to assess their approaches to building a local alliance, promoting IS, and engaging partners; and to note changes in the alliances over time,

#### Background

1. Alliance Name
2. Yrs
  a. 2019
  b. 2020
  c. 2021
  d. 2022
  e. 2023
3. Types of documents included
  a. Applications
  b. Final reports
  c. Other

#### Justification

4. Goals/objectives (already have this)
5. Why they wanted to do this

#### Activities

6. Capacity building (open text)
  a. Activity: approach, objectives, target audience, presenters, etc.
  b. Outcomes
7. Convenings (open text)
  a. Activity: approach, objectives, how often, etc.
  b. Outcomes:
8. Other Activities and Outcomes (open text)

#### Role of is

9. How IS was incorporated (open text)
  a. How activities supported or promoted IS
  b. Use of any TMF, outcomes, etc.
10. Where along the research continuum (open text)- development to dissemination

#### Engagement

11. Partners
  a. List of partners
    i. Youth (10–30 y.o.)
    ii. Family members
    iii. Clinicians/direct service providers
    iv. Policymakers federal
    v. Policymaker local
    vi. Program implementers
    vii. General Public
    viii. Funder
    ix. Other, please specify
  b. How/why they were selected (open text)
12. Engagement practices
  a. Engagement methods/strategies/activities (open text)
  b. Type of engagement- Pinto constructs
    i. Communication
    ii. Partnership exchange
    iii. Community capacity-building
    iv. Leadership
    v. Collaboration
  c. Timing of engagement (Asuquo, 2021)
    i. Pre-intervention phase referred to planning and readiness activities, including stakeholder advisory mechanisms, protocol development, ethical approval, field testing and related formative research activities.
    ii. Intervention phase referred to activities during the actual implementation of the HIV prevention intervention studies.
    iii. Post-intervention phase referred to dissemination, results reporting and related activities.

#### Changes over time

13. Note any shifts between applications (open text)
14. Note any shifts between application and final report (open text)
15. Sustainability efforts (open text)
